# Supplementary material for: A UMLS-based spell checker for natural language processing in vaccine safety
Source: BMC Med Inform Decis Mak. 2007 Feb 12;7:3. doi: 10.1186/1472-6947-7-3 (PMC1805499; doi:10.1186/1472-6947-7-3)
Supplement: Additional file 13 — RAP application directory. Sets up directory for RAP (RDF application for PHP) source code [file 1472-6947-7-3-S13.gz › rap/doc/phpdoc/__filesource/fsource_default__RdfAPI.php.html]

xml version="1.0" encoding="iso-8859-1"?


File Source for RdfAPI.php


# Source for file RdfAPI.php

Documentation is available at RdfAPI.php

```
1. <?php

3. // ----------------------------------------------------------------------------------
4. // RDF API for PHP
5. // ----------------------------------------------------------------------------------
6. // Version                   : 0.9.1
7. // Authors                   : Chris Bizer (chris@bizer.de),
8. //                             Radoslaw Oldakowski (radol@gmx.de)
9. // Description               : This file includes constatns and model package.
10. // ----------------------------------------------------------------------------------
11. // History:
12. // 08-09-2004                 : Packages added
13. // 08-06-2004                 : Class FindIterator added
14. // 11-19-2003                 : Class RdqlResultIterator added
15. // 07-31-2003                 : Classes N3Parser, N3Serializer added
16. // 07-27-2003                 : Classes RdqlParser, RdqlEngine, RdqlDbEngine, RdqlMemEngine added
17. // 06-25-2003                 : Class Model renamed to MemModel.
18. //                              Classes Model, DbModel, DbStore added.
19. //                              ADOdb Classes added
20. // 02-21-2003                  : Vocabularies and StatementIterator added.
21. // 09-15-2002                 : Version 0.1 of the API
22. // ----------------------------------------------------------------------------------


25. // Include Constants and base Object

27. require_once( RDFAPI_INCLUDE_DIR . 'constants.php' );
28. require_once( RDFAPI_INCLUDE_DIR . 'util/Object.php' );
29. include_once( RDFAPI_INCLUDE_DIR . PACKAGE_MODEL);


32. ?>
```

Documentation generated on Fri, 17 Dec 2004 16:16:52 +0100 by phpDocumentor 1.3.0RC3
